# TP53 Gene Polymorphisms and Occupational Skin Cancer Risks for Workers of Glass Fiber Manufacture

**Published:** 2017-11

**Authors:** Guzel F MUKHAMMADIYEVA, Denis O KARIMOV, Akhat B BAKIROV, Liliya K KARIMOVA

**Affiliations:** Ufa Research Institute of Occupational Health and Human Ecology, Ufa, Russia

**Keywords:** Occupational skin cancer, *TP53*, Polymorphism

## Abstract

**Background:**

Determining the role of genetic markers in individual sensitivity to chemical exposures raises a possibility of risk assessment of occupational diseases and their prevention. This paper focuses on the results of the identification of molecular-genetic markers associated with occupational skin cancer susceptibility. This study aimed to explore an association between polymorphisms of the *TP53* tumor suppressor gene and a risk of developing occupational skin neoplasms.

**Methods:**

This case-control study was conducted on 71 workers with occupational skin neoplasms, 99 healthy workers, and 100 healthy population-based controls in Bashkortostan Republic, Russia in 2015. Genotyping of *TP53* polymorphisms (rs1042522, rs1625895, and rs17878362) was performed by restriction fragment length polymorphism analysis of genomic DNA extracted from peripheral blood. Odds ratios and 95% confidence intervals were calculated to measure the strength of the association.

**Results:**

Subjects carrying allele C of rs1042522 were associated with an increased risk of occupational skin neoplasms [*P*=0.027, odds ratio (OR)=1.97, 95% confidence intervals (CI) 1.08–3.63]. An increased risk was also associated with allele 16bp of rs17878362 (*P*=0.010, OR=3.32, 95 % CI=1.31–8.78) and allele A of rs1625895 (*P*=0.003, OR = 5.45, 95 % CI = 1.72–19.15).

**Conclusion:**

The polymorphic variants rs1042522, rs1625895 and rs17878362 of the *TP53* gene are related to increased risks of occupational skin cancer. This study suggests the potential use of molecular-genetic data to assess increased individual risks of the development and prognosis of occupational skin neoplasms.

## Introduction

Non-melanoma skin cancer (NMSC) is the most common cancer among different population groups worldwide and its incidence is rising (1, 2). There are two main types of NMSC: squamous cell carcinoma (SCC) and basal cell carcinoma (BCC) (3). Important environmental and occupational risk factors for NMSC include physical agents (such as solar ultraviolet radiation, ionizing radiation), and chemical agents (such as arsenic and arsenic compounds, coal tars and pitches, mineral oils, shale oils, polycyclic aromatic hydrocarbons) (4–8). Workers exposed to certain chemicals over a long period in the metal-working, petroleum, agricultural industries and in glass factories, are at increased risk for occupational skin cancers (9–13).

In addition, predisposition to NMSC is mediated by genetic factors, including genetic alterations, caused by the total influence of multiple single nucleotide polymorphisms in the genes implicated in various molecular pathways (14–18).

The *TP53* gene located on chromosome 17p13.1 encodes a p53 tumour suppressor protein, involved in several important cellular processes, such as blocking the cell cycle, DNA repair, apoptosis induction and elimination of tumor cells. Genetic polymorphisms in *TP53* locus have been widely investigated for an association with skin cancer risks, but the results are inconsistent (19–21).

The study of polymorphic variants of the genetic systems regulating carcinogenesis, the identification of individual markers associated with malignancies, particularly among workers of carcinogenic industries, have acknowledged the importance of prevention and its corresponding level. Some progress is being made toward identifying trends in this field.

The studies were conducted among workers engaged in the manufacture of continuous glass fibers. These workers are known to be exposed to a combination of work environment factors, particularly carcinogens that are part of lubricants, glass fiber dust and heating microclimate along with micro-lesions of the skin, which have a pronounced toxic effect, manifested by the development of occupational skin neoplasms.

The aim of our study was to evaluate an association between polymorphisms (rs1042522, rs1625895, and rs17878362) of the *TP53* tumor suppressor gene and a risk of developing occupational skin neoplasms.

## Materials and Methods

### Subjects

The present case-control study was based on 270 Caucasian subjects divided into several groups in Bashkortostan Republic, Russia in 2015. Group I included 46 subjects with occupational limited hyperkeratosis; Group II - 25 patients with occupational skin malignancies. Group III comprised 99 skin disease-free workers engaged in the manufacture of continuous glass fiber. The latter was divided into two subgroups: workers with the length of employment up to 10 yr – 46 subjects and with the length of employment more than 10 yr – 53 subjects. Population-based control comprised 100 age- and ethnicity-matched healthy subjects without body contact with hazardous occupational factors and living in the same region. Findings of patients with occupational skin diseases were compared with those of Group III and population-based control.

After adjusting for age, sex, length of employment in hazardous work environment it turned out that Group I comprised 56.5% of males and 43.5% of females; in Group II there were 48.0% of males and 52.0% of females. There was prevalence of individuals aged 50–59 yr and 60–69 yr. The length of employment of all patients in Groups I and II was more than 10 yr.

Group III consisted of 47 females and 52 males, most of them were 30–39 and 40–49 yr of age. About 46.5% of workers had the length of employment up to 10 yr, 53.5% of workers had the 11 yr and more length of employment.

Biomedical Ethics Committee of the Ufa Research Institute of Occupational Health and Human Ecology approved the study. Blood samples were collected after obtaining informed consent from all participants.

### Genetic Analysis

For genetic studies, we used DNA samples isolated from lymphocytes of peripheral venous blood of workers by the method of phenol-chloroform extraction. The genotypes of *TP53* were determined by PCR-RFLP analysis. The primers, restriction enzymes, and PCR conditions for *TP53* were the same (22, 23).

### Statistical Analysis

Statistical analysis was performed using the SPSS (Chicago, IL, USA, Ver. 17). The significance of differences in frequency distributions of alleles and genotypes between groups was identified by comparing the sample using chi-square (χ^2^) test (with the Yates correction). The odds ratio (OR) and 95% confidence intervals (95% CI) were estimated. The results were considered statistically significant at *P*<0.05.

## Results

The results of distributions of genotypes and alleles of the polymorphic locus rs1042522 of the *TP53* gene in the studied groups are presented in Table 1.

**Table 1:** Frequency of alleles and genotypes of the polymorphic locus rs1042522 of the *TP53* gene in the groups studied

		***Number***	***Allele, n (%)***		***Genotype, n (%)***		
			***G***	***C***	***G/G***	***G/C***	***C/C***
Patients	Group I	46	59 (64.1)	33 (35.9)	17 (36.9)	25 (54.4)	4 (8.7)
	Group II	25	31 (62.0)	19 (38.0)	10 (40.0)	11 (44.0)	4 (16.0)
	Combined sample	71	90 (63.4) *#	52 (36.6) *#	27 (38.0)*	36 (50.7)	8 (11.3)
Healthy workers (Group III)	Length of employment up to 10 yr	46	54 (58.7)	38 (41.3)	14 (30.4)	26 (56.5)	6 (13.0)
	Length of employment more than 10 yr	53	82 (77.4)	24 (22.6)	31 (58.5)	20 (37.7)	2 (3.8)
Control		100	143 (75.3)	47 (24.7)	48 (48.0)	47 (47.0)	5 (5.0)

Significance of differences between healthy group workers with the length of employment more than 10 yr:

* - *P*<0.05; significance of differences between population-based control:

# - *P*<0.05

Accordingly, there was no evidence of differences in the distribution of genotypes of the polymorphic locus rs1042522 of the *TP53* gene in the combined sample of patients and in population-based control. However, there were statistically significant differences in the distribution of allele frequencies. Therefore, in the patient group there was a significant increase in the proportion of the C allele (36.6% compared to 24.7% in the control group), with the decreasing allele frequency G (63.4% vs 75.3% in the population-based control group) (χ^2^ = 4.93, *P*=0.027; OR=1.76; 95% CI 1.06–2.91).

Comparative analysis of the polymorphic locus rs1042522 of the *TP53* gene among patients and workers with a short length of employment did not show any statistical difference (*P*>0.05).

The differences in distributions of genotypes and alleles of the polymorphic locus rs1042522 of the *TP53* gene between sick and healthy workers with the length of employment more than 10 yr were revealed (Table 1), but statistical significance of these differences was achieved only in the combined sample of patients. We found a significant reduction in the frequency of homozygous genotype G/G to 38.0% in individuals with occupational skin neoplasms, compared with 58.5% in healthy workers (χ^2^ = 4.32, *P*=0.038; OR=0.44; 95% CI 0.20–0.96). The frequency of C allele in patients was significantly higher (χ^2^ = 4.94, *P*=0.027). The ratio of the odds equal to 1.97 points to the importance of risk alleles in the development of skin tumors of occupational origin (95% CI 1.08–3.63).

Comparative analysis of the frequency of alleles and genotypes for the polymorphic locus rs17878362 of the *TP53* gene between the combined sample of patients and the population-based control group revealed no significant differences (Table 2). The frequency of the 16bp allele was 19.0% in the patient group and 12.5% in the population control.

**Table 2:** Frequency of alleles and genotypes of the polymorphic locus rs17878362 of the TP53 gene in the groups studied

		***Number***	***Allele, n (%)***		***Genotype, n (%)***		
			***w***	***16bp***	***w/w***	***w/16bp***	***16bp/16bp***
Patients	Group I	46	75 (81.5)*	17 (18.5)*	30 (65.2)*	15 (32.6)*	1 (2.12)
	Group II	25	40 (80.0)*	10 (20.0)*	16 (64.0)*	8 (32.0)	1 (4.0)
	Combined sample	71	115 (80.9)**	27 (19.0)**	46 (64.8)*	23 (32.4)*	2 (2.8)
Healthy workers (Group III)	Length of employment up to 10 yr	46	70 (76.1)	22 (23.9)	24 (52.2)	22 (47.8)	0 (0)
	Length of employment more than 10 yr	53	99 (93.4)	7 (6.6)	46 (86.8)	7 (13.2)	0 (0)
Control		100	175 (87.5)	25 (12.5)	75 (75.0)	25 (25.0)	0 (0)

Significance of differences between the healthy group of workers and length of employment more than 10 yr:

* - *P*<0.05;

** - *P*<0.01

There were no significant differences in the frequency of alleles and genotypes of the polymorphic locus rs17878362 of the *TP53* gene between patient groups and worker groups with short length of employment.

The study of the polymorphic locus rs17878362 of the *TP53* gene showed the presence of significant differences in the genotype frequencies between the combined sample of patients with occupational skin neoplasms and healthy workers with the length of employment more than 10 yr (Table 2).

There was a significant reduction in the frequency of genotype w/w in the patient group to 64.8% vs. 86.8 % in the group of health workers (χ^2^ = 6.57, *P*=0.011). The Genotype w/w of the polymorphic locus rs17878362 of the *TP53* gene can be considered a protective factor regarding the development of occupational skin neoplasms (OR=0.28, 95% CI 0.10–0.77). There was an increase in the frequency of the heterozygous geno-type w/16bp in the combined sample of patients (32.4%) vs 13.2% in healthy workers with the length of employment more than 10 yr (*P*=0.025). In the patient group with occupational skin cancer, we found a statistically significant increase in the frequency of 16bp allele compared with healthy workers (χ^2^ = 6.89, *P*=0.010; OR=3.32, 95% CI 1.31–8.78).

Similar results were obtained when we compared the patient groups with different forms of occupational skin diseases and healthy workers having length of employment more than 10 yr.

The distribution findings on frequencies of geno-types and alleles of the polymorphic locus rs1625895 of the *TP53* gene in the patient group and in the population-based control are presented in Table 3. In the groups studied, significant differences were revealed only for the distribution of allele frequencies. Besides, in the combined sample of patients, more frequent was a statistically significant An allele (χ^2^ = 4.17, *P*=0.041; OR=2.04; 95% CI 1.03–4.05). Out of the patients studied, two subjects had the homo-zygous genotype A/A, which amounted to 2.8%, whereas in the population-based control group, this genotype was not detected.

**Table 3:** Frequency of alleles and genotypes of polymorphic locus rs1625895 of the *TP53* gene in the groups studied

		***Number***	***Allele, n (%)***		***Genotype, n (%)***		
			***G***	***A***	***G/G***	***G/A***	***A/A***
Patients	Group I	46	76 (82.6)**	16 (17.4)**	31 (67.4)**	14 (30.4)**	1 (2.17)
	Group II	25	41 (82.0)**	9 (18.0)**	17 (68.0)*	7 (28.0)*	1 (4.0)
	Combined sample	71	117 (82.4)**#	25 (17.6)**#	48 (67.6)**	21 (29.6)**	2 (2.8)
Healthy workers (Group III)	Length of employment up to 10 yr	46	70 (76.1)	22 (23.9)	24 (52.2)	22 (47.8)	0 (0)
	Length of employment more than 10 yr	53	102 (96.2)	4 (3.8)	49 (92.5)	4 (7.6)	0 (0)
Control		100	181 (90.5)	19 (9.5)	81 (81.0)	19 (19.0)	0 (0)

Significance of differences between the healthy group of workers with length of employment more than 10 yr:

* - *P*<0.05,

** - *P*<0.01; reliability of differences with population-based control:

# - *P*<0.05

Analysis of the polymorphic locus rs1625895 has failed to reveal significant differences (*P*>0.05) in the frequency of genotypes and alleles between the patients and health workers with the length of employment up to 10 yr (Table 3).

When comparing the patient groups and health workers with the length of employment more than 10 yr, significant differences in the distribution of genotypes and alleles of the polymorphic locus rs1625895 of the *TP53* gene have been revealed. In the combined sample of patients, the frequency of the heterozygous genotype G/A was 29.6%, whereas in healthy workers it accounted for 7.6% (χ^2^ =7.83, *P*=0.006). The frequency of the A allele of the polymorphic locus rs1625895 of the *TP53* gene was significantly higher in patients than in the healthy group (17.6%, and 3.8%, respectively, χ^2^ =9.95, *P*=0.003; OR=5.45, 95% CI 1.72–19.15). This fact indicates the importance of this risk allele in the development of occupational skin neoplasms.

## Discussion

In the present study, the association between the *TP53* polymorphisms and susceptibility to occupational skin neoplasms has been found. Increased risks of occupational skin neoplasms have been noted for allele C of rs1042522, allele 16bp of rs17878362 and allele An of rs1625895.

Single nucleotide polymorphism at codon 72 of exon 4 (rs1042522) is accompanied by substitution of arginine to proline, which causes arginine or proline p53 protein variants differing structurally and functionally. The arginine variant is more effective in inducing apoptosis.

The literature data on the association of the polymorphic locus rs1042522 of the *TP53* gene with risks of malignant neoplasms development are ambiguous and contradictory. In particular, the same allelic variants may have both protective and oncogenic effects depending on the geographical position of the population, ethnicity of individuals, localization and type of tumour, and exposure to hazardous environmental factors (24, 25). The association between polymorphism rs1042522 of the *TP53* gene and the development of melanoma in European populations has been noted. An increased risk of developing melanoma was associated with carriage of G/G genotype of the *TP53* gene (26). Meanwhile, studies on susceptibility to the development of epithelial skin cancers (basal cell carcinoma, squamous cell carcinoma) did not reveal any association between this polymorphism and the disease (14).

Polymorphism in non-coding region of the *TP53* gene (rs17878362) is characterized by duplication (repetition, or insertion) of 16 nucleotides in intron 3. The polymorphism rs17878362 of the *TP53* gene is associated with reduced expression of the gene and may be involved in the disruption of the activation processes of the target genes transcription required for the cell cycle arrest and apoptosis (27). Polymorphism in the intron 6 of the *TP53* gene (rs1625895) is presented by the replacement of guanine for adenine on the site of the restriction endonuclease MspI. This polymorphism is known to be able to alter the expression of protein p53. To our knowledge, no one study investigated the influence of these two polymorphisms on skin cancer. Nevertheless, a number of authors have shown the association of the minor alleles of the rs17878362 and rs1625895 polymorphisms with an increased risk of certain malignancies (28–32).

## Conclusion

The polymorphic loci rs1042522, rs1625895 and rs17878362 of the *TP53* gene may influence the risk of developing occupational skin neoplasms. It is imperative to include studies on molecular markers in the pre- and periodic physical examinations in order to detect individual sensitivity and prognostic risks of developing occupational diseases. Genetic studies must be conducted based on patients’ awareness, voluntary involvement, and confidentiality.

## Ethical considerations

Ethical issues (Including plagiarism, informed consent, misconduct, data fabrication and/or falsification, double publication and/or submission, redundancy, etc.) have been completely observed by the authors.
